# High Concentration of Serum Aspartate Aminotransferase in Older Underweight People: Results of the Kanagawa Investigation of the Total Check-Up Data from the National Database-2 (KITCHEN-2)

**DOI:** 10.3390/jcm8091282

**Published:** 2019-08-22

**Authors:** Michi Shibata, Kei Nakajima, Ryoko Higuchi, Taizo Iwane, Michiko Sugiyama, Teiji Nakamura

**Affiliations:** 1 School of Nutrition and Dietetics, Faculty of Health and Social Services, Kanagawa University of Human Services, 1-10-1 Heisei-cho, Yokosuka, Kanagawa 238-8522, Japan; 2 Department of Nutrition, School of Medicine, St. Marianna University, 2-16-1 Sugao, Miyamae-ku, Kawasaki, Kanagawa 216-8511, Japan; 3 Department of Endocrinology and Diabetes, Saitama Medical Center, Saitama Medical University, 1981 Kamoda, Kawagoe, Saitama 350-8550, Japan

**Keywords:** AST, ALT, aminotransferase, underweight, sarcopenia, body mass index, age

## Abstract

Background: Aspartate aminotransferase (AST) is pivotal in amino acid metabolism. However, the serum activity of AST, which leaks from multiple organs, including liver and skeletal muscle, is unknown in older underweight people, who are at high risk of skeletal muscle mass loss. Therefore, we measured the serum activities of AST and alanine aminotransferase (ALT), a liver-specific transaminase, in a large, community-based cross-sectional study. Methods: Clinical parameters and lifestyles were characterized in 892,692 Japanese people with a wide range of body mass indexes (BMIs; 13–39.9 kg/m^2^), aged 40–74 years old, who were undergoing a medical checkup. A general linear model was used to calculate the estimated mean of serum AST (EM-AST) in each BMI category after adjustment for confounding factors, including past history of cardiovascular disease and waist circumference. Results: Severe underweight (BMI 13–14.9 kg/m^2^) was present in 910 subjects (0.1%). Raw serum AST showed a J-shaped relationship with BMI, which was blunted in older subjects (60–74 years), and similar, but less curved relationships were identified for raw serum ALT and gamma-glutamyl transferase. These J-shaped relationships in serum AST were not altered when subjects were classified by sex, past history of cardiovascular and cerebrovascular diseases, and habitual exercise. EM-AST showed a U-shaped relationship, with a minimum at BMI 21–22.9 kg/m^2^, regardless of age and waist circumference. Conclusions: High serum AST but normal serum ALT is highly prevalent in older underweight people and might reflect skeletal muscle pathology.

## 1. Introduction

Serum aspartate aminotransferase (AST) activity is routinely measured worldwide as part of regular medical checkups because it can be rapidly, inexpensively, and reproducibly assayed. AST, a pyridoxal phosphate (the active form of vitamin B6)-dependent transaminase [1,2], is expressed in the liver, heart, skeletal muscle, kidney, brain, and red blood cells [3,4]. In general, the activity of alanine aminotransferase (ALT), a liver-specific transaminase, is measured simultaneously to distinguish hepatic and nonhepatic pathology [3,4,5,6]. These enzymes are important for amino acid degradation and biosynthesis, during which they catalyze the transfer of amino groups from aspartic acid or alanine to ketoglutaric acid to produce oxaloacetic acid and pyruvic acid, respectively [2,3]. Chronic malnutrition involves a shortage of vitamin B6 as well as other nutrients [7,8], which leads to dysfunction of these enzymes and multiple organ defects. To date, however, the serum activities of these enzymes have not been characterized in older underweight people, who are at a greater risk of skeletal muscle loss, which eventually results in sarcopenia and a higher mortality rate than that of normal-weight people [9,10].

Stable high serum AST activity accompanied by normal ALT can theoretically reflect widespread skeletal muscle and/or myocardial pathology [4,5,6], although defects in other organs, such as the kidney, and hemolytic anemia should be excluded [3,4,5,6]. In our preliminary study of 79,623 apparently healthy individuals [11], the serum activities of enzymes, including AST, alkaline phosphatase, and lactate dehydrogenase, were higher in older people with low body weight than in those with normal body weight or younger people with low body weight, resulting in a J-shaped relationship with body mass index (BMI), where serum AST fell in the low to normal BMI, then steeply rose above the level of serum AST in the lowest BMI across the increasing BMI. In contrast, serum ALT activity was consistently lower in individuals with low BMI of <19 kg/m^2^, regardless of their age. These results suggest a possibility that there may be widespread skeletal muscle pathology in older people with low body weight. However, in the preliminary study, severe underweight (BMI of <16 kg/m^2^ [12]) was not separately taken into consideration. In addition, relevant confounding factors, such as sex, smoking status, alcohol intake, physical activity level, and the activities of other enzymes were not adjusted for.

Therefore, in the present study, we measured the serum activities of AST and ALT and adjusted these values for relevant confounding factors in a larger community-based study of individuals with a wide range of BMIs (13–39.9 kg/m^2^).

## 2. Methods

### 2.1. Study Design

The present investigation was a cross-sectional study involving the secondary usage of Japanese health checkup data, termed the Kanagawa Investigation of Total Check-up Data from the National Database (KITCHEN), which involved sequential numbering and was aimed at elucidating the factors associated with cardiometabolic diseases. The fundamental study design is described in detail elsewhere [13]. Since 2008, everyone living in Japan who is aged 40–74 years is supposed to undergo a yearly specific health check, which is managed by the Japanese Ministry of Health, Labour and Welfare (MHLW) [14]. The study protocol was approved by the ethics committee of Kanagawa University of Human Services on 7 November 2016 (Approval number 10-43) and the Japanese MHLW on 19 December 2016 (Approval number 121). We received digitally recorded anonymous data regarding people living in Kanagawa Prefecture from the MHLW as part of its nationwide program, which involves the provision of medical data to third parties. To ensure that individuals could not be identified, their ages were categorized as 40–44, 45–49, 50–54, 55–59, 60–64, 65–69, or 70–74 years.

### 2.2. Subjects

Preexisting data collected from people aged 40–74 years who underwent a checkup between April 2008 and March 2009 in Kanagawa were analyzed. Disabled individuals who could not move without assistance and those residing in medical institutions, including hospitals and nursing homes, were not included. Subjects with a serum AST of ≥200 IU/L, an ALT of ≥200 IU/L, and/or a gamma glutamyl transpeptidase (GGT) of ≥300 IU/L were excluded. Subjects with a BMI of <13 kg/m^2^ were also excluded because this is the mean BMI for Japanese inpatients with anorexia nervosa [15,16]. Furthermore, subjects with a BMI of ≥40 kg/m^2^ were excluded because this is the threshold for class III obesity, which is very rare in the Japanese population (0.02%–0.07%) [13,17]. After these exclusions, 892,692 people (475,500 men and 417,192 women) for whom a complete dataset was available were enrolled in the study. The subjects were divided into three age groups: those in their 40s, those in their 50s, and those in their 60s or 70s (*n* = 288,134, 246,700, and 357,858, respectively). Subjects in their 60s were analyzed with those in their 70s because of the smaller number in their 70s (*n* = 110,500). To evaluate participant age as a single numeric value, we transformed the age groups (40–44, 45–49, 50–54, 55–59, 60–64, 65–69, and 70–74 years) into substituted ages corresponding to the median for each age group (42, 47, 52, 57, 62, 67, and 72 years, respectively).

### 2.3. Measurements of Clinical Parameters

Measurements were performed in the morning after an overnight fast. BMI was calculated as body mass (kg) divided by height squared (m^2^). Clinical biochemical parameters, including AST, ALT, and GGT activities, were measured alongside internal and external standards, as recommended by the MHLW [13]. Subjects were allocated to 13 BMI categories: 13–14.9, 15–16.9, 17–18.9, 19–20.9, 21–22.9, 23–24.9, 25–26.9, 27–28.9, 29–30.9, 31–32.9, 33–34.9, 35–36.9, and 37–39.9 kg/m^2^. Underweight and severe underweight were defined as <18.5 kg/m^2^ and <15 kg/m^2^, respectively [12]. High levels of serum AST and ALT were defined as ≥30 U/L for both parameters [3,6]. The ratio of AST/ALT activities was calculated because this is used to distinguish several disease processes [5,6]. Waist circumference (WC) was considered an important confounding factor because it correlates more closely with the degree of adiposity and metabolic abnormalities than BMI [18].

### 2.4. Statistical Analysis

Data are expressed as the mean ± standard deviation (SD) or median (interquartile range). Continuous and categorical variables were evaluated using analysis of variance (ANOVA) and the χ^2^-test, respectively. Differences in serum enzyme activities between age groups and between BMI categories were evaluated using the Bonferroni test. A general linear model and the least-square method were used to calculate the estimated means of serum AST (EM-AST) and ALT (EM-ALT), controlling for relevant confounding factors, including age, sex, smoking status, alcohol consumption, habitual exercise (≥30 min per session, at least twice weekly), physical activity (≥1 hour/day), pharmacotherapy for hypertension, diabetes, dyslipidemia, serum triglyceride, serum high-density lipoprotein (HDL) cholesterol, WC, serum GGT, and medical history of cardiovascular disease, without usage of stepwise procedure. In our study, the sample size of very severe underweight group (BMI 13–14.9kg/m^2^) was first calculated on the assumption of α, 1-β, SD, and effect as 5%, 80%, 10, and 2, respectively, to test the difference in means of serum AST, for instance, between old and young groups. Then, a total of around 800 subjects were needed for the severe underweight group. We also assumed that the prevalence of such population was equivalent to around 0.1% (0.06%–0.15%) in total according to our previous studies [11,13]. Therefore, the requisite whole size was calculated as around 800,000 subjects in our study.

Statistical analyses were performed using SAS-Enterprise Guide (SAS-EG 7.1) in the SAS system, version 9.4 (SAS Institute, Cary, North Carolina, USA). *p* < 0.05 was considered to represent statistical significance.

## 3. Results

The characteristics of the subjects, classified into three groups according to age, are shown in Table 1. There were 61,094 underweight and 910 severely underweight subjects (6.8% and 0.1% of the total, respectively). The prevalence of underweight decreased (7.5%, 6.4%, and 6.7%) with increasing age (χ^2^-test, *p* < 0.0001), whereas the prevalence of very severe underweight (BMI of 13–14.9 kg/m^2^) increased (*p* < 0.0001). The prevalence of subjects with high AST (≥30 U/L) increased with increasing age (*p* < 0.0001), whereas the prevalence of subjects with high ALT (≥30 U/L) decreased (*p* < 0.0001). As expected, most continuous variables and the prevalence of categorical parameters were higher in the older than in the younger groups (all *p* values < 0.0001 by ANOVA or χ^2^-test).

**Figure 1:**
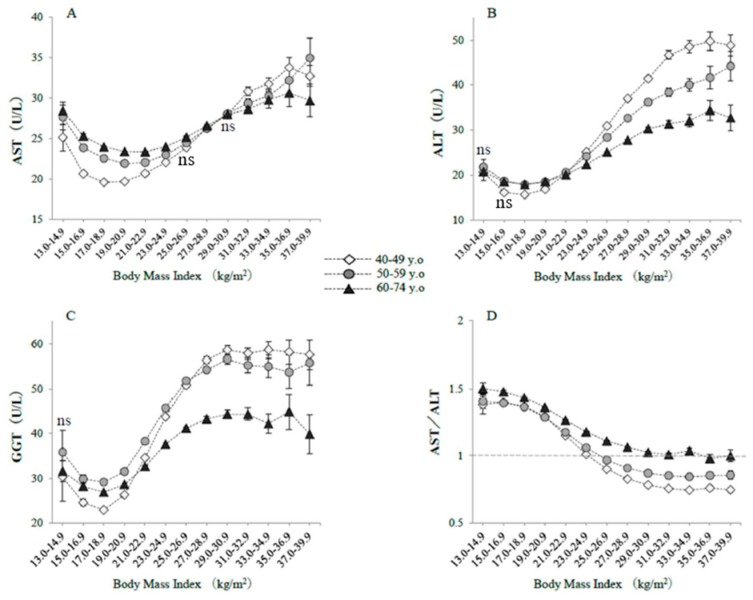
Raw serum enzyme activities, classified according to body mass index and age. The symbols indicate the mean values, and the vertical bars represent the 95% confidence intervals (CIs). BMI categories numbered 1–13 represent 13–14.9, 15–16.9, 17–18.9, 19–20.9, 21–22.9, 23–24.9, 25–26.9, 27–28.9, 29–30.9, 31–32.9, 33–34.9, 35–36.9, and 37–39.9 kg/m^2^. (**A**) AST, (**B**) ALT, (**C**) GGT, (**D**) AST/ALT ratios. The numbers of subjects in each BMI category were 185; 4028; 27,770; 60,097; 69,404; 56,582; 35,072; 18,206; 9054; 4268; 1950; 939; and 579 in their 40s; 198; 3332; 19,394; 45,814; 62,236; 55,433; 33,227; 15,689; 6741; 2712; 1168; 476; and 280 in their 50s; and 527; 5799; 27,686; 67,114; 96,657; 83,644; 46,035; 19,154; 7146; 2571; 944; 376; and 187 in the 60–74 year old category, respectively. The indication of ‘ns’ means no significant difference in values among three age groups in each BMI category. Otherwise, a significant difference was observed (ANOVA).

Figure 1 shows the raw serum enzyme activities, classified according to BMI category and age group. Serum AST was higher in both low-BMI and obese subjects, forming a J-shaped curve, particularly in subjects in their 40s, whereas the J-shaped relationship was blunted in subjects aged 60–74 years (Figure 1A). The raw serum AST activities in severely underweight subjects were significantly higher if they were older (28.5 ± 12.9 U/L) rather than in their 40s (25.1 ± 11.3 U/L) or if they had a BMI of 21–22.9 kg/m^2^ (23.4 ± 7.9 U/L) (all *p* values <0.0001, Bonferroni test). These differences were not affected if the serum AST values were logarithmically transformed. The raw serum ALT and GGT activities showed similar J-shaped relationships but with less extreme minima (Figure 1B,C). However, the serum activities of these two enzymes in the 60–74 year old group were not the highest, even for the underweight subjects. AST/ALT ratio was higher in older subjects and in subjects with lower BMI, with values >1 for all the BMI categories (Figure 1D).

**Figure 2:**
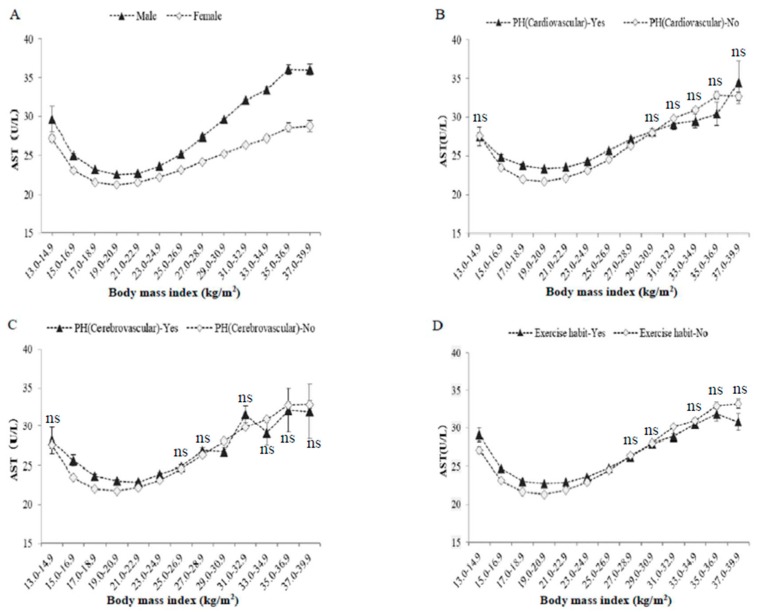
Raw serum AST activities, classified according to body mass index, sex, past histories, and exercise habit. The symbols indicate the mean values, and the vertical bars represent the 95% confidence intervals (CIs). BMI categories numbered 1–13 represent 13–14.9, 15–16.9, 17–18.9, 19–20.9, 21–22.9, 23–24.9, 25–26.9, 27–28.9, 29–30.9, 31–32.9, 33–34.9, 35–36.9, and 37–39.9 kg/m^2^. (**A**) Men and women, (**B**) past history of cardiovascular diseases, (**C**) past history of cerebrovascular diseases, (**D**) habitual exercise (≥30 min per session, at least twice weekly). The number of subjects in each BMI category was the same as in Table 1 and Figure 1. The indication of ‘ns’ means no significant difference in values between two groups in each BMI category. Otherwise, a significant difference was observed (ANOVA).

Figure 2 shows serum levels of AST in BMI categories according to sex, past history of cardiovascular/cerebrovascular diseases, and habitual exercise. Men had higher levels of serum AST than women throughout the BMI categories. Subjects with past history of cardiovascular disease, cerebrovascular diseases, or habitual exercise had higher levels of serum AST than those without in the nonobese BMI area (BMI <25 kg/m^2^). However, regardless of the classifications, all cases of serum AST showed J-shaped relationships against BMI, with a minimum around BMI of 19–20.9 kg/m^2^.

**Figure 3:**
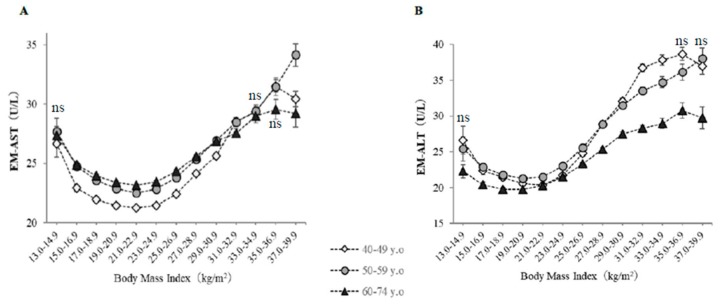
Estimated means of serum AST (EM-AST) and ALT (EM-ALT) activities, classified according to body mass index and age. The symbols indicate the estimated means of AST and ALT activities after controlling for the effects of age, sex, current smoking, daily alcohol intake, habitual exercise, physical activity, pharmacotherapy (for hypertension, diabetes, or dyslipidemia), history of cardiovascular disease, serum triglycerides, high-density lipoprotein cholesterol, GGT, and waist circumference. The vertical bars represent the 95% CIs. (**A**) EM-AST, (**B**) EM-ALT. The BMI categories numbered 1–13 are the same as in Table 1. The numbers of subjects are the same as in Figure 1. The indication of ‘ns’ means no significant difference in values among three age groups in each BMI category. Otherwise, a significant difference was observed (ANOVA).

Figure 3 shows EM-AST and EM-ALT, which were calculated using a general linear model, classified according to BMI category and age group. EM-AST, when corrected for potential confounding factors, including age, sex, smoking status, alcohol intake, habitual exercise, and physical activity, showed a U-shaped relationship with BMI, with a minimum at a BMI of 21–22.9 kg/m^2^, regardless of age (Figure 3A). EM-AST were higher in nonobese older subjects (BMI <25 kg/m^2^), whereas EM-ALT were lower in subjects with lower BMI and in older subjects after adjustment for confounding factors (Figure 3B).

## 4. Discussion

The present study was a large, community-based cross-sectional study of ~900,000 people, which enabled us to evaluate the prevalence of conditions according to BMI, even in severely underweight individuals, with adequate sample sizes and to adjust for many potential confounding factors. It is perhaps surprising that there were substantial numbers of individuals with BMIs of <15 kg/m^2^ that were eligible for the present study (*n* = 910, 0.1% of the total), despite the subjects being apparently healthy. Many studies have shown that serum AST and ALT activities increase with increasing BMI, which accompanies an increase in the prevalence of fatty liver [19,20]. However, the present study adds to the finding that serum AST activity is also high in people with lower BMI, especially older people, even after adjustment for potential confounders. Additionally, individuals with past history of cardiovascular and cerebrovascular diseases were enrolled in this study, but such conditions did not influence the J-shaped relationship between serum AST and BMI categories, although it is well known that damages of myocardium and skeletal muscle as well as neuromuscular disease often increase serum AST level [4,5,6]. The current results are consistent with our finding in a previous preliminary study conducted in a different population [11]. Previous studies have also shown that the activities of serum alkaline phosphatase and lactate dehydrogenase, which are also abundantly expressed in skeletal muscle [5,21,22], are significantly higher in older nonobese individuals. Taken together, these studies suggest that pathophysiologic conditions associated with high serum AST are present in older underweight people.

In contrast, serum ALT was normal or low in old subjects with lower BMI, implying that fewer old subjects had hepatic injury in the lower BMI group, despite the fact that fatty liver is often present in malnourished individuals [23,24]. Because all the subjects in the present study were apparently in good health and were able to attend the clinic or hospital for the checkup, acute inflammatory diseases, such as acute kidney disease or myocardial infarction, as well as severe chronic diseases, such as advanced liver cirrhosis, were unlikely to be present, even in the older underweight subjects. Instead, high AST, accompanied by normal or low ALT, predominantly reflects mild-to-moderate skeletal muscle pathology [5,6], which might imply the presence of incipient sarcopenia or frailty. However, further studies are required that use validated markers of skeletal muscle mass, such as creatine kinase, aldolase, or creatinine [5,25,26], or functional measurements, such as appendicular skeletal muscle index, mid-arm muscle circumference, grip strength, or 10 min gait time [10,27], to confirm or refute this hypothesis. The most plausible explanation for lower muscle mass in underweight individuals is the greater catabolism and/or lower synthesis of skeletal muscle protein resulting from energy depletion [28,29] or a deficiency of protein intake or essential amino acids, including branched-chain amino acids, that are required for muscle protein synthesis [9,10]. In addition, in the case of the elderly, this problem can be aggravated by complex geriatric syndrome, which involves several age-related factors, such as neuromuscular degeneration, changes in muscle protein turnover, changes in hormone secretion and sensitivity, chronic inflammation, oxidative stress, and behavioral or lifestyle factors [10]. Several sarcopenia biomarkers, including irisin, a myokine produced by muscle in response to exercise [30,31], have been considered for the last decade [32]. Although it has been shown that exercise training increased the expression of irisin in old people, it is unknown whether the expression of irisin is decreased in older underweight people.

It is noteworthy that the lowest EM-AST values were found in individuals with BMI of 21–22.9 kg/m^2^, which is the optimal BMI range for the Japanese population [33,34,35]. Additionally, high serum AST activity was also found in obese subjects, regardless of age, suggesting the presence of sarcopenic obesity, a combination of excess weight and reduced muscle mass, in a proportion of subjects [36,37]. However, it is unknown whether the mechanisms explaining high AST differ between underweight and obesity people.

During the last decade, several studies have shown that low serum ALT is associated with lower muscle mass [38], poor fitness [39], frailty [39,40], and sarcopenia [41]. Consistent with these, in the present study, the serum ALT was lower in subjects with lower BMI, especially in older subjects after adjustment for confounders. Therefore, presarcopenia and/or frailty may be more prevalent in underweight subjects. Indeed, individuals with mild-to-moderate anorexia nervosa, who are predominantly younger women, were likely to have been enrolled in the study. However, because many studies have shown that both serum AST and ALT activities are high in patients with anorexia nervosa [15,16], it is possible that, in fact, few individuals with anorexia nervosa were enrolled in this study.

Some limitations to the present study should be discussed. First, because of its cross-sectional nature, causality cannot be inferred. Second, we have not assayed validated markers of skeletal muscle and it may be that a combination of high serum AST and normal serum ALT activity does not accurately reflect widespread skeletal muscle pathology. Third, underweight individuals with subclinical malignancy, hyperthyroidism, infection, or chronic organ failure may have been included in the analyses [42,43], a possibility that should be addressed in future studies. Finally, the present results may not be applicable to other populations, such as those in western countries, who have higher BMIs than the Japanese population, which has a relatively low mean BMI.

## 5. Conclusions

Our study of ~900,000 general people has demonstrated that high serum AST is more prevalent in underweight individuals, especially older people, which may reflect widespread skeletal muscle pathology. This possibility should be investigated in further study, including the measurement of specific markers of skeletal muscle mass and function to assess sarcopenia and frailty.

## Figures and Tables

**Table 1:** Subjects’ characteristics classified according to age.

Age group (years old)	40–49	50–59	60–74
*n* (%)	288,134 (32.3)	246,700 (27.6)	357,858 (40.1)
Male, *n* (%)	173,096 (60.1)	140,837 (57.1)	161,567 (45.1)
Age (years)	44.3 ± 2.5	54.7 ± 2.5	66.8 ± 4
BMI (kg/m^2^)	22.9 ± 3.5	23.0 ± 3.3	22.8 ± 3.1
Waist circumference (cm) *	81.2 ± 9.6	82.5 ± 9.2	82.7 ± 8.9
Underweight BMI <18.5 (kg/m^2^), *n* (%)	21,526 (7.5)	15,654 (6.3)	23,914 (6.7)
Very severe underweight BMI <15 (kg/m^2^), *n* (%)	185 (0.06)	198 (0.08)	527 (0.15)
AST (U/L)	21.9 ± 8.9	23.2 ± 9	24.2 ± 9.1
ALT (U/L)	23.8 ± 17.2	23.4 ± 14.5	21.5 ± 12
AST/ALT ratio	1.06 (0.82–1.33)	1.08 (0.88–1.33)	1.19 (1.00–1.43)
AST ≥30 U/L, *n* (%)	33,573 (11.7)	33,153 (13.4)	53,993 (15.1)
ALT ≥30 U/L, *n* (%)	63,900 (22.2)	48,620 (19.7)	51,526 (14.4)
γ-Glutamyl transferase (U/L)	38.1 ± 37	41.5 ± 38.7	34.5 ± 31.5
Triglyceride (mg/dL)	88 (60–137)	97 (68–144)	99 (73–139)
High-density lipoprotein cholesterol (mg/dL)	63.5 ± 16.7	64.9 ± 17.5	64.3 ± 16.8
Pharmacotherapy for			
Hypertension, *n* (%)	13,203 (4.6)	37,634 (15.3)	112,871 (31.5)
Diabetes, *n* (%)	3479 (1.2)	8354 (3.4)	19,588 (5.5)
Dyslipidemia, *n* (%)	7376 (2.6)	19,279 (7.8)	66,228 (18.5)
Medical history Cardiovascular disease, *n* (%)	3520 (1.2)	7049 (2.9)	25,162 (7)
Medical history Cerebrovascular disease, *n* (%) **	1351 (0.5)	3087 (1.3)	13,456 (3.8)
Current smokers, *n* (%)	91,056 (31.6)	70,808 (28.7)	51,051 (14.3)
Alcohol drinking			
Every day, *n* (%)	82,992 (28.8)	79,796 (32.3)	92,531 (25.9)
Exercise (≥30 min per session) at least twice/week, *n* (%)	59,939 (20.8)	63,928 (25.9)	157,507 (44)
Physical activity (≥1 hour/day), *n* (%)	106,312 (36.9)	95,933 (38.9)	193,975 (54.2)

Data are presented as mean ± SD, median (interquartile range) or *n* (%). All differences in all continuous and categorical variables between the three age groups were significant (all *p* values < 0.0001, according to ANOVA or the χ^2^-test). * Available *n* = 889,512 total. ** Available *n* = 891,681 total. AST, aspartate aminotransferase; ALT, alanine aminotransferase, BMI, body mass index.
